# Overall Exposure of European Adult Population to Mycotoxins by Statistically Modelled Biomonitoring Data

**DOI:** 10.3390/toxins13100695

**Published:** 2021-10-01

**Authors:** Barbara De Santis, Francesca Debegnach, Piero Toscano, Alfonso Crisci, Paola Battilani, Carlo Brera

**Affiliations:** 1 Department of Food Safety, Nutrition and Veterinary Public Health, Italian National Institute of Health, 00161 Rome, Italy; francesca.debegnach@iss.it (F.D.); carlo.brera@iss.it (C.B.); 2 Institute of BioEconomy, National Research Council, 50145 Florence, Italy; piero.toscano@ibe.cnr.it (P.T.); alfonso.crisci@ibe.cnr.it (A.C.); 3 Department of Sustainable Crop Production, Università Cattolica del Sacro Cuore, 29122 Piacenza, Italy; paola.battilani@unicatt.it

**Keywords:** mycotoxin, biomarker, exposure assessment, provisional daily intake, biomonitoring

## Abstract

This study presents the exposure scenario to mycotoxins of adult population throughout Europe. The urinary biomarkers values were obtained by modelling data from two European projects. Exposure to AFB1, OTA, CIT, FBs, DON, NIV and T2/HT2 are presented. The main output obtained refers to a concern for public health about AFM1, FBs, T2/HT2 and NIV, and low concern for OTA, DON and CIT. The margin of exposure for AFM1 did not respect the reference value of 10,000 considered of low priority for risk; for *Fusarium* toxins, FBs and T2/HT2, probable daily intake (PDI) values resulted about ten times higher than their tolerable daily intake and NIV presented the most critical situation with a calculated PDI 30 times higher than the reference TDI value. North and South Europe scenarios were also depicted by clustering biomonitoring data. OTA and DON showed to be prevalent in Northern countries and the opposite was noticed for ZEN, higher in Southern countries. The critical issues of the availability of records feeding the dataset and of the accuracy of excretion rate for some mycotoxins are source of uncertainty for the reliability of the outputs, nevertheless the time is ripe for asking for more concrete HBM values and/or HBM-HBGV which would help in interpreting the burden of mycotoxins in Europe.

## 1. Introduction

Food safety has to be considered by all the stakeholders as a pre-requisite characterizing any product along the whole food chain, from raw materials to processed products.

Contaminants are among the factors that can affect food safety. They may enter the food chain at any stage, from primary production to consumption; it is essential to keep them at toxicologically acceptable levels in the interest of public health protection, in other words “safe for public health” [1].

For being safe, a food product has to contain doses of contaminants lower than the corresponding health-based guidance values and/or factorial indicators such as the margin of exposure (MoE) for genotoxic carcinogenic compounds [2].

The approaches used for calculating the exposure and characterizing the risk are well defined and straightforward when only one contaminant is present in a food product [3]. This is based on the intrinsic toxicological effect of the compound, the existence of a toxicological end-point, the contamination level, the consumption rate of the product and the body weight of the examined consumer group.

More challenging is to assess the exposure and the corresponding toxic effects when in a food product co-occur several contaminants, both belonging to the same class of compounds, i.e., more than one mycotoxin co-occurring, and to different ones, e.g., co-presence of mycotoxins and heavy metals. In this case, relevant gaps still exist such as the lack of a fixed toxicological end-point and the type of toxic effect, e.g., synergistic and/or additive. Further, there are numerous sources of uncertainties affecting the quantitative determination of the overall metabolic pathway of the parent compounds and their combination in PBTK (physiologically based toxicokinetic) modelling, a very limited amount of information on xenobiotics co-occurrence both in all crops and at a single sample level, and quite scarce amount of reliable quantitative data on the toxicity of mixtures of chemical compounds, including toxicokinetic and toxicodynamic [4].

Mycotoxins are food and feed contaminants naturally occurring in a wide spectrum of plant and animal origin food products [5]. They are produced by fungi mainly belonging to *Aspergillus*, *Penicillium* and *Fusarium* genera. Diverse fungi can co-occur in a crop; each fungus can produce one or more native mycotoxins, but modified forms can also be detected. This was recently emphasized by climate change, with extreme events faced during the growing season [6].

The assessment of the exposure and the risk characterization of the resulting mixture represent a challenging task. Among the main critical points, the development of prioritization criteria of mycotoxin mixtures to be tested.

The calculation of dietary intakes of chemical compounds, as single or in mixtures, has to be performed by handling the existing contamination data on food products composing the dietary habit. Unfortunately, the heterogeneous distribution of mycotoxins in food plays a key role in assessing the actual contamination levels, making the assessment of the exposure by the diet challenging and requesting a big effort to gather proper consumption data. Performing human biomonitoring (HBM) studies by analyzing the biological fluids and tissues for testing the presence of the internal dose of a single or multiple mycotoxins (or their metabolites), is an essential tool for exposure assessment that, in combination with occurrence data, can give more complete information to manage risk assessment and support the outputs [7,8].

In the context of mycotoxins, as well as other xenobiotics, biomonitoring studies consider the overall exposure to a chemical hazard while providing information on the variability and trends in exposure scenarios. The use of biomarker (BM) measurements for trying to connect the internal with the external dose should count on (i) suitable/validated PBTK models to properly quantify the biotransformation, metabolism and excretion of all the BMs compounds, (ii) applicable mode of action (MOA) or adverse outcome pathways (AOP) toxicological frameworks to describe biological key events leading to an effect, (iii) available flexible approaches to multiple chemicals, (iv) structured schemes of biological fluid sampling and related analysis and (v) the validation of the identified BMs of exposure or effect. At present, despite various studies performed in vitro on the combined toxicological effects of mixtures of mycotoxins, this information is far from being considered exhaustive. The assessment of the exposure to multiple mycotoxins is commonly based on data available in literature on the co-occurrence of mycotoxins in specific crops and/or food products; they are clustered, adopting an appropriate algorithm for calculating the exposure and characterizing the risk on the basis of theoretical assumptions. On the other hand, if it is a fact that biomonitoring studies on multiple mycotoxin biomarker are available, the assessment of the exposure to multiple mycotoxins contamination is complex and the scientific community is making efforts to deal with numerous gaps for combining data and for interpreting and depicting risk assessment scenarios of exposure to single mycotoxins.

From the above, the aim of this paper is to present the attempt to assess the exposure to mycotoxins by using already published BM data. For targeting the objective, a probabilistic methodology was adopted using the goodness-of-fit method (KS-gof tests) to overcome the initial biomarkers data scarcity, to assess the uncertainty and provide a reliable estimation of the BM concentration. Biomarker data from two research projects, “Mycotoxin mixtures in food and feed: holistic, innovative, flexible risk assessment modelling approach: MYCHIF” [9] and “Experimental study on deoxynivalenol biomarkers in urine—DONEXPO” [10] was used. Finally, the modelled biomarker concentrations were used as input for the exposure assessment to single mycotoxins, calculated by adopting the approach of the probable daily intake (PDI). Several exposure scenarios have been selected depending on body weight and mycotoxin biomarker concentration class, namely mean, lower bound (LB) and upper bound (UB); the exposure has been assessed also clustering scenarios by geographical area (North vs. South Europe).

## 2. Results and Discussion

### 2.1. Dataset

From the combined collection of biomarker data (MYCHIF and DONEXPO data are available at http://mychifrep.fi.ibimet.cnr.it/, data extraction and reconstruction, covering the period 2010–2017, are summarized in Table 1. In Table 1 the number of records and samples are reported, the first referring to the number of cohorts/groups for each study, while the latter to the total number of samples. All results of the KS-gof tests (D parameter and *p*-value) are reported in Tables S1–S3, in the Supplementary Materials. The distributions showing the lowest D value and a significant *p*-value (*p*-value < 0.05) were adopted to refuse the null hypothesis of normal distribution and to calculate the final biomarker concentration for PDI scenario simulation. Among 14 mean concentration scenarios (5 in S1, 3 in S2 and 6 in S3), Weibull distribution returned the best statistical performance in terms of D and p-value for more than 85% of the scenarios (12/14), while in the remaining cases (2/14) the normal distribution (Norm) prevailed both in terms of D and p-value. For the concentration scenario that contemplate the LB class of biomarkers (11 in total), the Norm (6/11) tends to prevail over Weibull (3/11 scenario) and exponential (Exp) (2/11 scenario). Finally, for the concentration scenario with class UB of biomarkers (in total 11) there is no clear prevalence of one distribution over the others: indeed, Weibull returned the best results in 5/11 scenario, Exp 4/11 and Norm 2/11. Focusing on mycotoxin, only for OTA the same type of distribution (Weibull) was obtained for all the scenario under consideration; for all the other mycotoxins, the distributions vary with the concentration class considered. In general, the prevalence of the Weibull (20/36 scenario) and Norm (10/36 scenario) distribution is in line with what is reported in the literature; indeed, these two distributions tend to better represent life data, especially Weibull is very flexible in fitting empirical data and is frequently used within reliability engineering and risk analysis [11,12].

**Figure 1:**
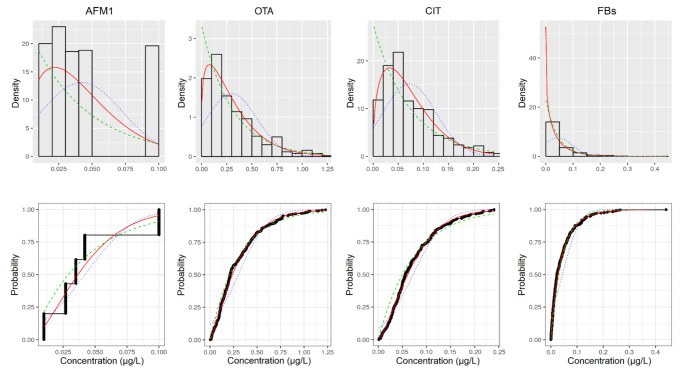
Histogram of the empirical bootstrapped data and theoretical fitting distribution for AFM1, OTA, CIT and FBs (panels **above**). Cumulative distribution function of the empirical bootstrapped data and theoretical fitting distribution for AFM1, OTA, CIT and FBs (panels **below**). Empirical bootstrapped data (black histogram and black circle); Exp function (dashed green line); Norm function (dots blue line); Weibull function (solid red line).

**Figure 2:**
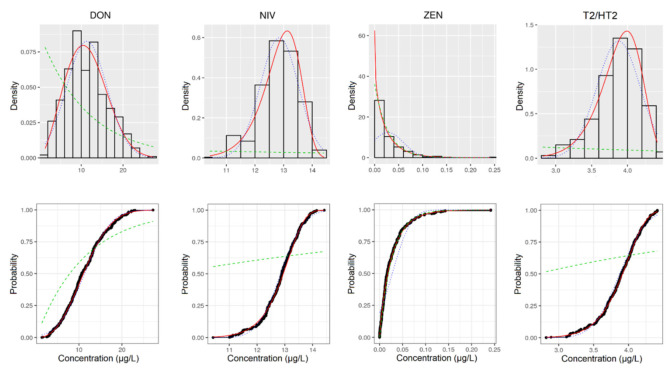
Histogram of the empirical bootstrapped data and theoretical fitting distribution for DON, NIV, ZEN and T2/HT2 (panels **above**). Cumulative distribution function of the empirical bootstrapped data and theoretical fitting distribution for DON, NIV, ZEN and T2/HT2 (panels **below**). Empirical bootstrapped data (black histogram and black circle); Exp function (dashed green line); Norm function (dots blue line); Weibull function (solid red line).

Detailed fitting plots in terms of density plot and cumulative distribution function (CDF) are presented in Figure 1 for aflatoxin M1 (AFM1), ochratoxin A (OTA), citrinin (CIT) and fumonisins (FBs), and in Figure 2 for deoxynivalenol (DON), nivalenol (NIV), zearalenone (ZEN) and T2 and HT2 toxins (T2/HT2). For each plot empirical bootstrapped data and theoretical distribution were reported. The distribution showing the most elevated degree of significance (Tables S1–S3) was taken for the workflow second step.

### 2.2. PDI Calculation

#### 2.2.1. European Scenario

The PDI outputs for adults (male and female) European scenario with P50 body weight are shown in Table 2, all the countries in Europe, which produced eligible data for each mycotoxin, were accounted. The reported PDI values for OTA, FBs, DON, ZEN and T2/HT2, accounted for more than seven records, while for AFM1, CIT and NIV, less than seven records were present in the dataset. Each PDI value (mean, LB and UB) was obtained by the Equation (1), using the P50 body weight, extracted from the EFSA comprehensive data base [13], and the biomarker value that each distribution gave as the best average value in each concentration class (mean, LB and UB). The confidence interval associated is a measure of the reliability of the results, confirming the powerfulness of the simulated distribution. The PDI outputs for all weight scenarios (P5-P50-P95) are shown in Tables S4–S7.

For risk assessment purposes, the obtained PDI values were compared with each of the health based guidance values (HBGV) (e.g., tolerable daily intake—TDI) established by EFSA. For those mycotoxins with no threshold for toxicological effects, due to their genotoxic and carcinogenic activity, the HBGV approach is not applicable [2] and the established reference point (e.g., benchmark dose lower limit—BMDL) and the estimated PDIs were considered to assess the margin of exposure in the risk characterization. The MoE approach is only suggestive of a level of concern taking into account additional uncertainties.

The estimated PDI for DON and ZEN, produced values below the established TDIs (1 and 0.25 µg/kg bw/day for DON and ZEN, respectively) [14,15]. In particular, the upper bound exposure estimated for DON is 2.5 folds lower than the TDI; for ZEN, this value is about four folds lower than the reported TDI, indicating that, in case of adult consumers, there is not a public health concern for these mycotoxins. This is in agreement with the dietary European exposures carried out by EFSA for these mycotoxins [14,16]. As regards FBs, NIV and T2/HT2, the estimated PDIs values were compared with the set TDIs of 1, 1.2 and 0.02 µg/kg bw/day, respectively, [17,18] and an exceedance of the fixed values is observed for these mycotoxins. In particular, FBs and T2/HT2 mean UB PDI values are about ten times higher than their TDIs, reflecting a scenario of health concern. However, the PDI_LB mean values are in agreement with the mean LB dietary exposure values that EFSA published in 2014, which at that time were considered below the former established TDI of 2 µg/kg bw/day. It has to be noted that for FBs the LB and UB range (0.17597–19.6532 µg/kg bw/day) is extremely wide, accounting for a high number of left-censored data.

The worst scenario was obtained for NIV, with a mean PDI that is almost 30 folds higher than the reference TDI. An additional comment to be performed on the uncertainty around NIV and PDI, regards the excretion rate (ER) that, as reported in the literature, is derived from animal studies exclusively [19], therefore weighting to the uncertainty of the estimation. Moreover, the number of BM data available was critically low and coming from only two countries.

The estimated PDIs for AFB1 and OTA were compared with BMDL10 established by EFSA [20,21] and CIT PDI was compared with the level of no concern for nephrotoxicity derived by the NOAEL (no observed adverse effect level) established by EFSA [22].

As regards AFB1, starting from the AFM1 modelled data, the MoE was calculated comparing the BMDL10 of 0.4 µg/kg bw per day with the PDI. The calculated MoE value of 6 is far below the value of 10,000, revealing a concern/criticism for public health. As known, for substances of no-threshold effects, values higher than 10,000 correspond to low concern for public health [2]. However, under the ALARA principle applied to either genotoxic or carcinogenic compounds, aflatoxins risk assessment is automatically of potential concern. Urinary AFM1 is a validated biomarker for aflatoxins exposure; however, the data feeding the dataset were limited (less than seven records scenario and only two countries represented) and were modelled with an empirical plot of data that tended to replicate the original pattern, weakening the representativity of the scenario.

As regards OTA, the recently reviewed EFSA scientific opinion [21] updated the HBGV establishing a BMDL10 of 4.73 µg/kg bw/day for non-neoplastic effects (calculated from kidney lesions observed in pigs). Upon this non-neoplastic endpoint, the MoE ranged from 14 to 48 for LB and UB estimation, respectively, indicating a health concern when compared with the reference value of 200 reported by EFSA [21]. The BM data available for OTA is well represented with a good numerosity of records giving consistency to the obtained scenario.

For CIT, no TDI is reported and the calculated PDI was compared with a level of no concern for nephrotoxicity in humans of 0.2 µg/kg bw/day [22]. In particular, for risk assessment purpose, the MoE approach was carried out also for CIT and the resulting value is 21. Because of the lack of toxicological information, genotoxicity and carcinogenicity could not be excluded for this mycotoxin, therefore the MoE approach takes a reference value of 10,000, below which a concern is underlined. As already pinpointed for AFM1 and NIV, an uncertainty in the estimation has to be considered for the poor representativity of CIT biomarker for data numerousness and country of origin of the published data (one country).

As a general comment, it is important to look at the obtained results keeping in mind all the limitation included. With the exclusion of DON, for which a consistent number of data were derived from a single project (DONEXPO), the data collected from literature are not homogeneous despite the tentative harmonization. All the considered mycotoxins are not supported by the same amount of data, there is a variability in the sampling criteria, and BM analysis may have differences in terms of validation, BM stability, and internal/external quality assessment of the laboratory producing BM data. Extensive HBM studies have been conducted on AFM1, OTA and FBs, DON and ZEN and multiple evaluations of the related HBGVs or BMDLs have been carried out through the years, so data are available. Other mycotoxins such as CIT, NIV and T2/HT2 are much less represented in the BM studies, so the few data available should be taken with the proper caution. In addition, with respect to excretion rate values, the scenario is not homogeneous for the different mycotoxins. Extensive studies on the fate of ingested DON have been published, generating accurate and reliable excretion rate, also supported by the general agreement of the values reported in the literature [23,24].

As previously cited, the mycotoxin concentration data reconstructed reflect urinary biomarkers, however, while AFM1, OTA, DON and FB1 in urine are all biomarkers of exposure that have been validated, for CIT, NIV, ZEN and T2/HT2 the association with the diet is still under study, and, hopefully, with the impulse that biomonitoring is receiving in the last years, there will be soon more reliable information for these and other mycotoxins.

#### 2.2.2. North/South Europe Scenarios

PDI outputs simulated for adults North/South Europe scenarios for P50 body weight are shown in Table 3. The PDI was calculated for the three biomarkers available for both areas, namely OTA, DON and ZEN, considering three PDI classes of concentration, mean, LB and UB. The adults’ body weight is calculated as average of all European countries included in the North or South scenario. The PDI outputs for all weight scenarios (P5-P50-P95) are shown in Tables S8–S10.

The comparison of the obtained PDI values shows higher exposures for the North scenario for OTA and DON with PDIs that are almost three and two times higher than in the South region, respectively. Conversely, for ZEN the South scenario produced a PDI that is about 35 times the one estimated for the North region.

Without disregarding the uncertainties highlighted, as hypothetical explanations for the observed differences in PDIs, the different dietary habits in the two different geographical regions could also be counted. As far as OTA, a higher urine levels reported in Northern regions may reflect higher consumption of typical potential OTA contaminated foods such as beer, pork meat products and coffee [25]. DON is a field mycotoxin produced by various *Fusarium* genotypes, *Fusarium graminearum* and *culmorum* widespread in Europe [26] commonly occurring in a small grain cereal. The difference may reflect the known geographical susceptibility with the shift of northern countries to be affected by the *F. graminearum*, and with a dietary habit to consume higher variety of diverse small grains (e.g., rye and oat), preferably whole grain products instead of refined wheat-based products more typical in Southern countries. The opposite trend observed for ZEN could not be interpreted by the same considerations requiring further investigations.

As already mentioned for the European scenario, OTA and PDIs are compared with the BMDL10 of 4.73 µg/kg bw/day to end up with a calculated MoE ranging from 25 for South and 7 for North, respectively. Hence, comparing MoEs with the reference of 200 [21], both the South and North scenario should be recognized as a priority for management actions since a concern cannot be excluded.

The DON PDI upper bound value compared with the TDI (1 µg/kg bw/day) confirm that there is a sufficient protection assurance being half of the TDI for the North Europe scenario and four times lower than the TDI for the South Europe scenario.

For ZEN, the South scenario produced a mean PDI that is about 35 times the one estimated for the North region. To note that BM values gathered for South are driven especially by the data coming from Spain, which reveal a specific exposure scenario. However, as a general result the comparison with the TDI reveals a protective scenario (22% of the TDI and <1% of the TDI, for South and North, respectively). It has to be noted that these conclusions are limited to the papers published in the period 2010–2017, and a more recent study by Ali and Degen [27] reports ZEN concentrations generally higher than those used for this PDI calculation.

### 2.3. Moving Forward

The biomonitoring tool for mycotoxins exposure is promising tool and the scientific community is making efforts to combine data for interpretation and depiction of risk assessment scenarios of exposure. The potential of HBM is recognized and a number of biomonitoring studies on mycotoxins have been produced worldwide. In Europe, various research groups have produced measures of biomarkers in different biospecimens, but still, harmonization is far and there are several issues to be resolved in order to make the biomonitoring tool strong enough to be used as a reliable assessment tool at European scale. Toxicokinetic parameters and analytical issues make difficult to ensure that biomonitoring in specimen appropriately reflects the exposure and that the variability of concentrations is not only a matter of analytical weakness. The comparison among biomonitoring results should be performed under the assumption that samplings were carried out taking into consideration sources and timing of exposure appropriately defined upon the target mycotoxin. However, the use of available BM data needs to be considered.

Certainly, the dataset presented in this paper includes important limiting gaps. Besides the standardization of analytical procedures and the definition of the quality assurance systems, the biomarker data strongly depends upon appropriate limits of quantification and/or limits of detection; moreover, data available are always aggregated which make their use limited. So, together with the scarce availability, also the quality of data represents a limit for their use. The non-homogenous quality of data represented a bottleneck to feed the dataset, and the auspice is that the availability of more harmonized, reliable and accurate measurements is comprehensively reached. In these regards, efforts are being performed; the European project HBM4EU [28] is trying to reach key objectives including the creation of a European platform for the biomonitoring by harmonizing analytical procedures, coordinating sampling rules, generating scientific evidence between human exposure to chemicals and health, also adapting chemical risk assessment methodologies to use human biomonitoring data. Mycotoxins have been included in the priority list of the project so that an acceleration is expected in the systematic revision of all the gaps and in the definition of an appropriate workflow.

Concomitantly, a priority for future perspective is the definition of HBM health related guidance values or biomonitoring values able to guide the risk assessment in interpreting the BM data. The extensive work carried out for other contaminants (heavy metals or pesticides) where years of long experience has been accumulated, could be used as a guidance. On this regard the work carried on by the German Human-Biomonitoring Commission [29] for the definition of HBM-HBGV for environmental contaminants, inspired the idea to describe and define the same parameters also for mycotoxins. In fact, by back calculating the value from the HBGV or reference points defined and revised by EFSA (TDIs, BMDLs or NOAELs) and using established toxicokinetic extrapolations (e.g., ER) [29], these HBM-HBGVs could be derived for mycotoxins. The German HBM Commission proposed the approach for the definition of HBM-HBGV values, where BM-I is the value considered as an equivalent level at which no risk of health effect exists. The BM-I represents a provisional concentration level of a substance in human biological material at which, and below which, no concern for a risk of adverse health effect is expected. BM-I can be considered the starting point. At the same time, whenever a known biomarker concentration has been associated with an adverse health effect, thus representing a value linked with a health concern, a BM-II value can be defined. For all the values in between, a health effect cannot be excluded [29]. At present, for BM-II no mycotoxin BM values have been associated with human health effect and neither epidemiology has shed light on the prevalence, incidence or cause of health effect; on the contrary, a BM-I value could be established if all the parameters are available (i.e., TDI, urinary excretion factor and volume of urine).

DON may be taken as a pilot example to this purpose. Urinary DON is a validated biomarker and has been extensively studied, thus is the most represented in the dataset records. The excretion rate parameter is reliable and in good agreement (on the percentage figures) among the different studies [24,30]. Taking DON TDI (1 μg/kg bw/day) and the excretion rate (72%, Table 5), and using the volume of urine per kg bw (2 L/kg bw/day) we can obtain a BM-I of 21.56 μg/L for adults as a reference. Comparing this reference value with what was obtained from the models applied in this study (mean value of 10.07 μg/L and a LB-UB range of 5.994–10.34 μg/L), it can be concluded that the adult exposure to DON in Europe fits into a non-concern zone.

A further critical point to highlight is the lack of consolidated approaches to assess the cumulative risk for mycotoxins for the risk characterization. For the cumulative risk assessment different methods are available [3] and are applied for different chemical mixtures in human, animal and ecological area. The main problem formulation arises for the kind of interaction effects among toxicants in the mixtures, the dose response data and the criteria for the grouping. In the case of the toxicants that act independently and have a similar mode of action, by assuming dose-addition criteria, the hazard index (HI) is applied. The HI metrics already applied in a number of studies for comparing predicted exposure to a reference point or a reference value, has been successful especially for environmental chemicals [31,32] and for pesticides [33]. Unfortunately, since mycotoxins have different mode of actions and affect different target organs, the HI approach is not applicable; instead, the point of departure index (PODI) may be used for the assessment of potential risk. The effect-addition criteria are more suitable for mycotoxins and the PODI may be calculated as the sum of the exposure of each compound divided by its respective point of departure (POD being the BMDL or NOEL), corrected by an equivalency factor, if appropriate. In MYCHIF project an attempt was carried out [34] but the approach still needs a proper support and validation from the scientific community of toxicologist and risk assessors to be approved. This means that much more discussion is needed and more should be developed for the benefit of the risk managers to interpret in routine risk scenario with flexible, transparent tools.

## 3. Conclusions

In conclusion, this study presents the perspective of the internal mycotoxin measurement used as a biomarker to link the biomonitoring with mycotoxin intake. While being aware of the heterogeneity of the data, a dataset of measured BMs were statistically modelled to obtain the suitable distribution to be used to represent scenarios of exposure in Europe. The availability of records feeding the dataset, the heterogeneity of the data, the accuracy of excretion rate for some mycotoxins are source of uncertainty for the reliability of the outputs, nevertheless the time is ripe for asking for more concrete HBM value and/or HBM-HBGV which would help in interpreting the burden of mycotoxins in Europe. Therefore, this study is launched also with the aim to cope with the weakness and emphasize the strength of the approach.

In this study, European adults’ exposure to single mycotoxins calculated by a PDI approach was assessed. The main evidences refer to a concern for public health about AFB1, OTA, FBs, T2/HT2 and NIV and low concern for all the other investigated mycotoxins. For AFB1 and OTA, the calculated MoE was much lower than the margins considered to be in the area of no concern; for *Fusarium* toxins, FBs and T2/HT2 PDI values resulted to be about ten times higher than their TDIs and NIV presented the most critical picture with a calculated PDI higher than the corresponding HBGV value.

Regarding the comparison of the trends of exposures between South and North European countries, OTA and DON showed to be more present in Northern countries and vice versa ZEN exposure was found to be higher in Southern countries.

Notwithstanding the caution in the conclusive outputs, considering the numerousness of data, the limited number of countries feeding the dataset for some biomarkers and the uncertainty in the ER values available in the literature, the output of European scenarios presented is considered a valuable initial exercise evocative of adults’ exposure obtained by a statistical model which gave remarkable results.

## 4. Materials and Methods

### 4.1. Dataset

The biomarker dataset employed for human exposure estimation was collected in two EFSA projects, “Mycotoxin mixtures in food and feed: holistic, innovative, flexible risk assessment modelling approach: MYCHIF” and “Experimental study on deoxynivalenol biomarkers in urine—DONEXPO”.

Within the MYCHIF project [9], extensive literature searches (ELS) were undertaken for data collection on different topics including biomonitoring studies on mycotoxins of major toxicological relevance to humans and target animal species in all the possible specimen. The ELS performed for the purpose of the project covered the period 2010–2018 and initially retrieved a total of 5753 records that were submitted to the inclusion/exclusion criteria step. From these, only studies reporting biomarker data on human urine in adults were selected. Urine is a relatively accessible and manageable biological fluid; it represents the majority of the collected data of the whole dataset and for this biological substrate quite a number of mycotoxin biomarkers are available. Additional inclusion/exclusion criteria were fixed to select healthy European adults. Moreover, only mean and max values with standard deviation information were accepted and data with concentration values, irrespective of the units used, were included. A number of studies published for the purpose of the method setting/validation containing biomarker measurements, was also considered. The acceptance/rejection criteria for the dataset compilation included the availability of critical method performance parameters, such as LODs (limit of detection) and LOQs (limit of quantification) that are considered of crucial importance in the biomonitoring field, to substantiate the reliability of the method. After the selection criteria, the final number of references on human studies summed up 176 articles, that produced more than 2500 records of mycotoxin biomarker values. The biomarker dataset generated for this study covered the period 2010–2017. The number of publications on DON was the majority, followed by OTA, ZEN and FBs. In general, quite a few publications dealing with other mycotoxins (i.e., T2/HT2 toxins, AFM1, NIV and CIT) have been scrutinized. As regards the concentration levels in the final biomarker dataset, for aflatoxins, only data on AFM1 were included; data on OTA itself were selected for the dataset, while for CIT the sum of CIT and DH-CIT was included. As regards FBs the sum of FB1 and FB2 was considered; the DON values are reported in the dataset as total DON (the sum of DON-glucuronides and free DON); for NIV only data on the parent compound were reported; for zearalenone, ZEN, α-zearalenol and β-zearalenol were summed [35] and expressed as ZEN; similarly, T2 and HT2 were summed, and the biomarker concentration was reported as T2.

The DONEXPO data [10], reporting only total DON levels measured in urine samples collected in Italian, Norwegian and UK population in 2014, was also used to feed the dataset of the present study. Participants were recruited covering different age ranges and a ratio of 1:1 male/female. Of the total records that were produced within the DONEXPO project, the adults’ data (male and female) accounting for 395 raw data (267 female and 128 male) were selected and added to the ones from the MYCHIF reviewed literature. Although the DONEXPO report was included in the papers retrieved by the ELS of MYCHIF, it was decided to use the available raw data instead of mean values to feed the dataset.

### 4.2. Data Clustering

**Figure 3:**
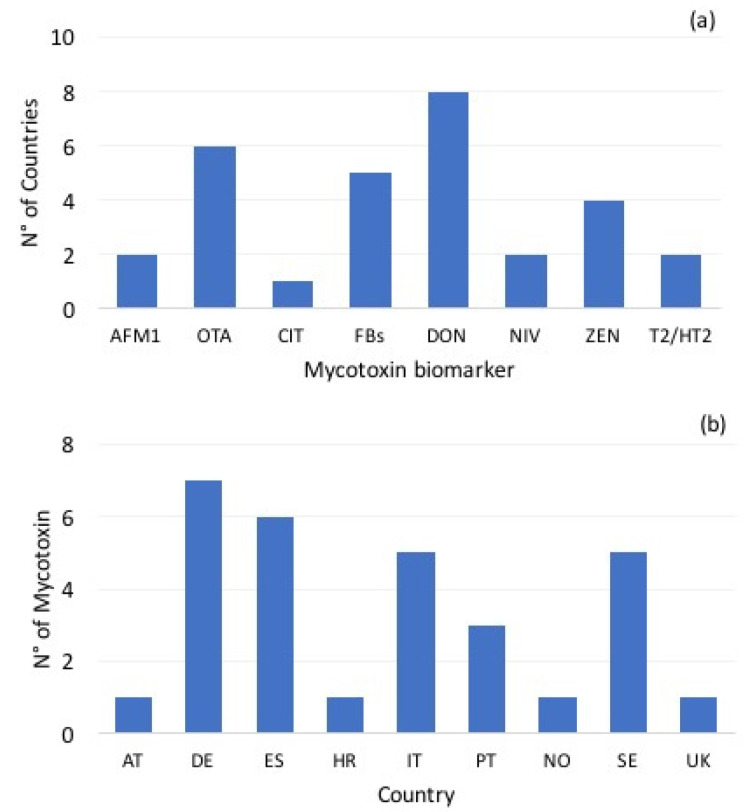
(**a**) Number of countries feeding each mycotoxin biomarker; (**b**) number of mycotoxins present in each country biomarker dataset.

In this paper, the whole dataset consisted of a number of urine biomarkers of different mycotoxins found in European healthy adults. A first clustering was carried out on the basis of the numerosity of records available. The first group included mycotoxin biomarkers with a numerosity above 7 records and comprised a scenario with OTA, FBs, DON, ZEN and T2/HT2 toxins. A second group included mycotoxin biomarkers with a numerosity below 7 records in the dataset and AFM1, CIT and NIV biomarkers were taken into account. For the first group, the PDI was calculated taking into account mean, LB and UB values, and for the second group only mean values were used for data cluster; three body weights scenarios (percentile 5—P5; percentile 50—P50; percentile 95—P95) were calculated from the body weights extracted from EFSA comprehensive data base for each country in the cluster [13]. According to the number of countries feeding each mycotoxin biomarker and the number of mycotoxins present in each country biomarker dataset, as shown in Figure 3, the exposure calculations were performed on the European perspective, neglecting the single country exposures.

A second clustering was based on a geographic criterion, considering data availability, both in terms of biomarkers and of country distribution. To characterize the PDI in the macro-areas scale and to evaluate any possible difference among them for the dietary habits [13] and peculiar climatic conditions [36], the geographic clustering was performed along two areas, the South Europe scenario, which included Italy, Portugal, Spain and Croatia, and the North Europe scenario, which included Austria, Germany, Norway, United Kingdom and Sweden. For these two geographic clusters the PDI was calculated for the three biomarkers available for both areas, namely OTA, DON and ZEN, considering three PDI classes of concentration, namely mean, LB and UB.

### 4.3. Statistical Analysis

Starting from biomarker dataset generated in this study (MYCHIF plus DONEXPO), a work-chain, to assess the PDI scenario levels for several mycotoxins widely affecting European population, was provided.

A two-step workflow was implemented, the first step aimed to provide biomarkers information through the study of a robust parametric distribution concerning biomarker data classes, namely LB, mean and UB; when original dataset numerosity was below 7 (records < 7), only the mean value class was considered.

Only records reporting concentration values (data at sample level) or mean values (aggregate data) were extracted. Values lower than the limit of detection (LOD) or lower than the limit of quantification (LOQ) were set to −1 if <LOD, and −2 if <LOQ. Subsequently, since these data were used for robust parametric distribution analysis and then to calculate PDI for exposure assessments in humans, these were treated by the substitution method [37] so that, at the LB scenario all results reported as lower than the LOD were set to zero and to the numerical value of the LOD for results reported as lower than the LOQ; at mean (MEAN) scenario, the results below the LOD were set to LOD/2 and to LOQ/2 for results reported as lower than the LOQ; at the UB, the results below the LOD were set to the numerical value of the LOD and to the value of the LOQ for results below the LOQ.

Generally, BM data have a similar form to survivor/lifetime data and often show exponential-like behavior. A very recent approach in biomarker data fitting, involves flexible size distributions as “generalized beta distribution” [38] that nests many well-known distributions (exponential, two parameter Weibull, lognormal and others). In this work, three simple continuous distributions for data fitting were adopted, namely, the two parameters Weibull (2par-WEIB) that has a scale (α) and shape (γ) parameter, the exponential defined by a single mean parameter (μ) and the normal distribution defined by a mean (μ) and a standard deviation (SD) parameter. In this work, the different numerosity among mycotoxin biomarkers data was harmonized by resampling for achieving information about data distribution by skewness-kurtosis plots (*Pearson plots*) of bootstrapped samples (N = 500).

All data samples (N = 500) were bootstrapped and processed by using the “fitdistrplus” R package [39]. The goodness of fit was evaluated by using the Lilliefors-corrected Kolmogorov–Smirnov goodness-of-fit test available in the “KSCorrect” R package [40]. This test works to identify the reliable fitting distribution when parameters are unknown and is measured by D parameter. It is possible to understand the meaning of D as a measure of the maximum vertical distance between the empirical cumulative distribution function (ECDF) of the sample and the cumulative distribution function (CDF) of the reference distribution.

### 4.4. PDI Calculation

To assess the PDI, the formula proposed by Solfrizzo et al. was used:

PDI (μg/kg bw/day) = (C × V × 100)/W × ER(1)

where C is the biomarker concentration (μg/L), V is the mean daily urine production (L) (for adults 2 L according to EFSA [41]), W is the mean body weight (kg) (as P5, P50 or P95, extracted from the EFSA comprehensive data base [13]) and ER is the biomarker excretion rate (%).

Biomarker data used in the scenarios are generated by the probabilistic methodology and summarized in Table 4.

The excretion rate values for all the selected mycotoxins were derived from the literature. For AFB1, the average of the extrapolated values of urinary AFM1 excreted by male (range 1.23–2.18%) and female (range 1.30–1.78%) was taken for back calculation of AFB1 exposure [42]. For OTA, the 2.5% of the urinary excretion derived by the approximation of the renal clearance of radioactivity carried out by Studer-Rohr in 2000 was taken for the assessment [43,44]. The urinary excretion rate of 40.2%, accounting for the summed contribution of CIT and DH-CIT, was derived by the median value obtained in the trial of Degen and co-authors [45]. As regards FB1, a value of 0.3% was taken as a mean value of FB1 urinary excretion rates ranges (0.075% and 0.5%) reported in literature [46,47]. The ER of 72% was considered for DON according to Turner et al. [48]. In the absence of human data, a value of animal urinary excretion rate of 1% was the best approximation value available for NIV [19]. An ER of 36.8% calculated for piglet, was considered for ZEN excretion in accordance with Gambacorta et al. [49]. Finally, for T2 and its metabolite, an average value of pig urinary excretion rate of 20% was the best approximation value for T2 exposure assessment [18]. The adopted excretion rate values are listed in Table 5.

## Figures and Tables

**Table 1:** Overall research dataset summary. The number of records (NR), the number of samples (NS), the mean (Mean) and the standard deviation (SD) are reported for each mycotoxin biomarker (μg/L) and for each country. Countries are reported as ISO 3166-1 alpha-2 country code.

Toxin	Parameter	AT	DE	ES	HR	IT	NO	PT	SE	UK
AFM1	NR/NS		1/50			4/169				
	Mean/SD		0.01/-			0.04/0.02				
OTA	NR/NS		7/104	6/242	1/40	1/52		20/1150	1/252	
	Mean/SD		0.63/1.04	0.40/0.28	0.93/1.13	0.06/0.31		0.02/0.004	0.46/0.57	
CIT	NR/NS		3/30							
	Mean/SD		0.172/0.027							
FBs	NR/NS		1/50	1/27		3/157		1/68	1/252	
	Mean/SD		0.005/-	2.25/0.35		0.06/0.03		2.5/0.0	0.007/0.004	
DON	NR/NS	1/27	8/281	6/87		126/403	149/298	2/11	5/1155	129/516
	Mean/SD	0/2.4	3.51/3.48	8.45/12.3		5.62/5.79	6.66/5.04	10.8/7.78	2.77/1.43	21.09/27.47
NIV	NR/NS			5/60					1/252	
	Mean/SD			15.44/1.83					0.02/0.74	
ZEN	NR/NS		4/80	6/87		7/356			1/252	
	Mean/SD		0.02/0.013	1.03/0.44		0.05/0.03			0.03/0.006	
T2/HT2	NR/NS		1/101	6/87						
	Mean/SD		0.04/-	3.95/6.21						

**Table 2:** PDI values calculated for adults’ European scenario, the calculations were performed with the P50 body weight value. In parenthesis, the lower and upper confidence levels are reported.

Mycotoxin	PDI_Mean (μg/kg bw/Day)	PDI_LB (μg/kg bw/Day)	PDI_UB (μg/kg bw/Day)	TDI (μg/kg bw/Day)
OTA ^1^	0.2878	0.0988	0.3494	4.73 ^3^
	(0.2772–0.2984)	(0.0964–0.1012)	(0.3436–0.3550)	
FBs ^2^	8.99929	0.17597	19.65327	1
	(8.98985–9.00872)	(0.17036–0.18158)	(19.21803–20.08852)	
DON ^1^	0.38846	0.23349	0.41134	1
	(0.38507–0.39185)	(0.22973–0.23726)	(0.40335–0.41933)	
ZEN ^2^	0.03483	0.00143	0.06532	0.25
	(0.03479–0.03487)	(0.00139–0.00147)	(0.06256–0.06807)	
T2/HT2 ^1^	0.16885	0.15962	0.20799	0.02
	(0.16859–0.16911)	(0.15401–0.16524)	(0.20258–0.21341)	
AFB1 ^1^	0.06385			0.4 ^3^
	(0.06101–0.06668)			
CIT ^1^	0.009484			0.2 ^4^
	(0.009172–0.009796)			
NIV ^1^	35.79271			1.2
	(35.75385–35.83157)			

^1^ Obtained by two parameters Weibull continuous distribution. ^2^ Obtained by normal distribution. ^3^ BMDL10. ^4^ Level of no concern for nephrotoxicity.

**Table 3:** PDI values calculated for adults’ South and North Europe scenarios, the calculations were performed with the P50 body weight value. In parenthesis the lower and upper confidence levels are reported.

Mycotoxin	PDI_Mean (μg/kg bw/Day)	PDI_LB (μg/kg bw/Day)	PDI_UB (μg/kg bw/Day)	Area
OTA ^1,2^	0.6764	0.6764	0.6764	North Europe
	(0.6662–0.6866)	(0.6662–0.6866)	(0.6662–0.6866)	
DON ^1^	0.48282	0.27699	0.53178	North Europe
	(0.4796–0.48603)	(0.27162–0.28236)	(0.51759–0.54597)	
ZEN ^1^	0.00149	0.00145	0.00159	North Europe
	(0.00146–0.00152)	(0.00143–0.00148)	(0.00156–0.00162)	
OTA ^1^	0.1874	0.1746	0.2024	South Europe
	(0.1844–0.1906)	(0.1688–0.1806)	(0.1954–0.2092)	
DON ^1^	0.24309	0.19531	0.25879	South Europe
	(0.23905–0.24712)	(0.19156–0.19907)	(0.25378–0.26380)	
ZEN ^1^	0.05553	0.05552	0.10588	South Europe
	(0.05551–0.05556)	(0.05550–0.05555)	(0.10380–0.10795)	

^1^ Obtained by two parameters Weibull continuous distribution. ^2^ No needs for data reconstruction, only mean class is available.

**Table 4:** Modeled mycotoxin biomarker concentration (μg/L), data used as input for PDI calculation. Concentrations are provided for all the three classes (mean, LB and UB). For mean concentration class, lower and upper confidence interval are also provided.

Mycotoxin	Scenario	Concentration (Mean; μg/L)	Lower Confidence Interval (Mean; μg/L)	Upper Confidence Level (Mean; μg/L)	LB Concentration (μg/L)	UB Concentration (μg/L)
AFM1	European	0.03871	0.03692	0.04050	NA	NA
OTA	European	0.26559	0.25601	0.27517	0.08794	0.31446
CIT	European	0.13687	0.13237	0.13978	NA	NA
FBs	European	0.96978	0.96876	0.97080	0.01849	2.09468
DON	European	10.07178	9.98468	10.15888	5.94282	10.34675
NIV	European	12.83527	12.82103	12.84950	NA	NA
ZEN	European	0.46020	0.45964	0.46077	0.01905	0.88318
T2/HT2	European	3.63374	3.62790	3.639577	3.56309	4.41715
OTA	North Europe	0.61299	0.60379	0.62219	0.61299	0.61299
DON	North Europe	12.67093	12.58648	12.75538	7.39707	13.94056
ZEN	North Europe	0.01958	0.01920	0.01996	0.01821	0.02145
OTA	South Europe	0.16489	0.16204	0.16773	0.15423	0.173492
DON	South Europe	6.12992	6.02877	6.23107	4.92539	6.54026
ZEN	South Europe	0.71473	0.71445	0.71502	0.01749	1.36606

**Table 5:** Biomarker concentration (μg/L), modeled data used as input for PDI calculation.

Mycotoxin	Excretion Rate (%)	Reference
AFM1	1.6225	[42]
OTA	2.5	[43,44]
CIT	40.2	[45]
FBs	0.3	[46,47]
DON	72	[23]
NIV	1	[19]
ZEN	36.8	[49]
T2 toxin	60	[18]

## Data Availability

Data is contained within the article and Supplementary Materials.

## Supplementary Materials

The following are available online at https://www.mdpi.com/article/10.3390/toxins13100695/s1. Table S1: Lilliefors-Corrected Kolmogorov–Smirnov Goodness-of-Fit Test results for the model implementation for European scenario for OTA, FBs, DON, ZEN and T2/HT2 mycotoxins with original size dataset ≥ 7 records. In bold the model chosen based on the lowest D and a significant *p*-value (*p*-value < 0.05); Table S2: Lilliefors-Corrected Kolmogorov–Smirnov Goodness-of-Fit Test results for the model implementation for European scenario for AFM1, CIT and NIV mycotoxins with original size dataset < 7 records. In bold the model chosen based on the lowest D and a significant *p*-value (*p*-value < 0.05); Table S3: Lilliefors-Corrected Kolmogorov–Smirnov Goodness-of-Fit Test results for the model implementation for North Europe and South Europe scenarios for OTA, DON and ZEN mycotoxins with original size dataset ≥ 7 records. In bold the model chosen based on the lowest D and a significant *p*-value (*p*-value < 0.05); Table S4: PDI results for AFM1, CIT and NIV. Three weights scenario were implemented only for MEAN concentration class. Lower and upper confidence interval of PDI were also reported; Table S5: PDI results for OTA, FBs, DON, ZEN and T2/HT2 using LB concentration class and three weights scenario. Lower and upper confidence interval of PDI were also reported; Table S6: PDI results for OTA, FBs, DON, ZEN and T2/HT2 using MEAN concentration class and three weights scenario. Lower and upper confidence interval of PDI were also reported; Table S7: PDI results for OTA, FBs, DON, ZEN and T2/HT2 using UB concentration class and three weights scenario. Lower and upper confidence interval of PDI were also reported; Table S8: PDI results for OTA, DON and ZEN using LB concentration class, three weights scenario for North and South Europe. Lower and upper confidence interval of PDI were also reported; Table S9: PDI results for OTA, DON and ZEN using MEAN concentration class, three weights scenario for North and South Europe. Lower and upper confidence interval of PDI were also reported; Table S10: PDI results for OTA, DON and ZEN using UB concentration class, three weights scenario for North and South Europe. Lower and upper confidence interval of PDI were also reported.
