# Supplementary material for: Overall Exposure of European Adult Population to Mycotoxins by Statistically Modelled Biomonitoring Data
Source: Toxins (Basel). 2021 Oct 1;13(10):695. doi: 10.3390/toxins13100695 (PMC8537926; doi:10.3390/toxins13100695)
Supplement: Supplementary file 1 [file toxins-13-00695-s001.zip › updated toxins-1360791-supp final.pdf]

Supplementary Materials: Overall Exposure of European Adult Population to Mycotoxins by Statistically Modelled Biomonitoring Data

Barbara De Santis, Francesca Debegnach, Piero Toscano, Alfonso Crisci, Paola Battilani and Carlo Brera

Table S1. Lilliefors-Corrected Kolmogorov-Smirnov Goodness-of-Fit Test results for the model implementation for European case study for OTA-FBs-DON-ZEN-T2/HT2 mycotoxins with original size dataset ≥ 7 records. In bold the model chosen based on the lowest D and a significant *p*-value (*p*-value < 0.05).

Mycotoxin	Case Study	Concentration Scenario	Weibull D	Weibull *p*-value	EXP D	EXP *p*-value	NORM D	NORM *p*-value
OTA	Adults_EU	LB	**0.204**	**0.001**	0.272	0.001	0.251	0.001
FBs	Adults_EU	LB	0.246	0.065	0.442	0.001	**0.326**	**0.002**
DON	Adults_EU	LB	0.221	0.001	0.117	0.001	**0.113**	**0.001**
ZEN	Adults_EU	LB	0.311	0.001	0.452	0.001	**0.240**	**0.001**
T2/HT2	Adults_EU	LB	0.469	0.001	0.762	0.001	**0.404**	**0.001**
OTA	Adults_EU	Mean	**0.050**	**0.003**	0.094	0.001	0.141	0.001
FBs	Adults_EU	Mean	0.050	0.002	0.572	0.001	**0.046**	**0.014**
DON	Adults_EU	Mean	**0.050**	**0.005**	0.284	0.001	0.078	0.001
ZEN	Adults_EU	Mean	0.050	0.002	0.560	0.001	**0.044**	**0.022**
T2/HT2	Adults_EU	Mean	**0.050**	**0.003**	0.540	0.001	0.040	0.057
OTA	Adults_EU	UB	**0.180**	**0.001**	0.395	0.001	0.328	0.001
FBs	Adults_EU	UB	0.260	0.054	0.552	0.001	**0.372**	**0.001**
DON	Adults_EU	UB	0.154	0.001	**0.115**	**0.001**	0.268	0.001
ZEN	Adults_EU	UB	**0.219**	**0.001**	0.477	0.001	0.329	0.001
T2/HT2	Adults_EU	UB	0.223	0.076	0.395	0.003	**0.338**	**0.002**

Table S2. Lilliefors-Corrected Kolmogorov-Smirnov Goodness-of-Fit Test results for the model implementation for European case study for AFM1-CIT-NIV mycotoxins with original size dataset < 7 records. In bold the model chosen based on the lowest D and a significant *p*-value (*p*-value < 0.05).

Mycotoxin	Case Study	Concentration Scenario	Weibull D	Weibull *p*-value	EXP D	EXP *p*-value	NORM D	NORM *p*-value
AFM1	Adults_EU	mean_only	**0.050**	**0.003**	0.324	0.001	0.314	0.001
CIT	Adults_EU	mean_only	**0.050**	**0.002**	0.134	0.001	0.130	0.001
NIV	Adults_EU	mean_only	**0.050**	**0.006**	0.569	0.001	0.046	0.014

Table S3. Lilliefors-Corrected Kolmogorov-Smirnov Goodness-of-Fit Test results for the model implementation for North Europe and South Europe case studies for OTA-DON-ZEN mycotoxins with original size dataset >= 7 records. In bold the model chosen based on the lowest D and a significant *p*-value (*p*-value<0.05).

Mycotoxin	Case Study	Concentration Scenario	Weibull D	Weibull p-value	EXP D	EXP *p*-value	NORM D	NORM *p*-value
OTA	Adults_North Eu	LB	**0.244**	**0.014**	0.400	0.001	0.290	0.002
OTA	Adults_South Eu	LB	**0.300**	**0.001**	0.489	0.001	0.329	0.001
DON	Adults_North Eu	LB	0.174	0.001	**0.120**	**0.001**	0.130	0.001
DON	Adults_South Eu	LB	0.237	0.001	0.209	0.001	**0.152**	**0.001**
ZEN	Adults_North Eu	LB	0.160	0.443	**0.262**	**0.049**	0.157	0.459
ZEN	Adults_South Eu	LB	0.398	0.001	0.646	0.001	**0.369**	**0.001**
OTA	Adults_North Eu	Mean	**0.050**	**0.004**	0.118	0.001	0.134	0.001
OTA	Adults_South Eu	Mean	**0.050**	**0.001**	0.063	0.003	0.150	0.001
DON	Adults_North Eu	Mean	**0.050**	**0.003**	0.334	0.001	0.060	0.001
DON	Adults_South Eu	Mean	**0.050**	**0.001**	0.086	0.001	0.143	0.001
ZEN	Adults_North Eu	Mean	**0.050**	**0.001**	0.332	0.001	0.318	0.001
ZEN	Adults_South Eu	Mean	**0.050**	**0.008**	0.605	0.001	0.053	0.002
OTA	Adults_North Eu	UB	**0.244**	**0.010**	0.400	0.001	0.290	0.002
OTA	Adults_South Eu	UB	**0.300**	**0.001**	0.516	0.001	0.329	0.001

DON	Adults_North Eu	UB	**0.128**	**0.001**	0.136	0.001	0.262	0.001
DON	Adults_South Eu	UB	0.195	0.001	**0.170**	**0.001**	0.195	0.001
ZEN	Adults_North Eu	UB	0.151	0.531	**0.311**	**0.023**	0.138	0.662
ZEN	Adults_South Eu	UB	0.254	0.001	**0.243**	**0.022**	0.174	0.081

Table S4. PDI results for AFM1-CIT-NIV. Three weights scenario were implemented only for MEAN concetration class. Lower and upper confidence interval of PDI were also reported.

MYCOTOXIN	WEIGHT	PDI MEAN	PDI LOWER CONFIDENCE INTERVAL	PDI UPPER CONFIDENCE INTERVAL
AFM1	59.9	0.07966	0.07598	0.08335
CIT	59.9	0.011368	0.010994	0.011742
NIV	59.9	42.85565	42.80811	42.90318
AFM1	71.8	0.06385	0.06101	0.06668
CIT	71.8	0.009484	0.009172	0.009796
NIV	71.8	35.79271	35.75385	35.83157
AFM1	86.29	0.05396	0.05170	0.05622
CIT	86.29	0.007891	0.007632	0.00815
NIV	86.29	29.77674	29.74410	29.80938

Table S5. PDI results for OTA-FBs-DON-ZEN-T2/HT2 using LB concetration class and three weights scenario. Lower and upper confidence interval of PDI were also reported.

MYCOTOXIN	WEIGHT	PDI LB	PDI LOWER CONFIDENCE INTERVAL	PDI UPPER CONFIDENCE INTERVAL
OTA	59.9	0.1182	0.1154	0.1210
FBs	59.9	0.20931	0.20261	0.21602
DON	59.9	0.27832	0.27383	0.28280
ZEN	59.9	0.00176	0.00171	0.00180
T2/HT2	59.9	0.19504	0.18826	0.20182
OTA	71.8	0.0988	0.0964	0.1012
FBs	71.8	0.17597	0.17036	0.18158
DON	71.8	0.23349	0.22973	0.23726
ZEN	71.8	0.00143	0.00139	0.00147
T2/HT2	71.8	0.15962	0.15401	0.16524
OTA	86.29	0.0810	0.0792	0.0830
FBs	86.29	0.14371	0.13901	0.14842
DON	86.29	0.19136	0.18830	0.19442
ZEN	86.29	0.00122	0.00119	0.00126
T2/HT2	86.29	0.13201	0.12738	0.13663

Table S6. PDI results for OTA-FBs-DON-ZEN-T2/HT2 using MEAN concetration class and three weights scenario. Lower and upper confidence interval of PDI were also reported.

MYCOTOXIN	WEIGHT	PDI MEAN	PDI LOWER CONFIDENCE INTERVAL	PDI UPPER CONFIDENCE INTERVAL
OTA	59.9	0.3474	0.3350	0.3600
FBs	59.9	10.7797	10.7683	10.7910
DON	59.9	0.46827	0.46421	0.47233
ZEN	59.9	0.04174	0.04169	0.04179
T2/HT2	59.9	0.20230	0.20199	0.20262
OTA	71.8	0.2878	0.2772	0.2984
FBs	71.8	8.99929	8.98985	9.00872
DON	71.8	0.38846	0.38507	0.39185
ZEN	71.8	0.03483	0.03479	0.03487
T2/HT2	71.8	0.16885	0.16859	0.16911
OTA	86.29	0.2384	0.2300	0.2468
FB	86.29	7.48895	7.48104	7.49687
DON	86.29	0.32519	0.32237	0.32801
ZEN	86.29	0.02900	0.02897	0.02904
T2HT2	86.29	0.14065	0.14043	0.14087

Table S7. PDI results for OTA-FBs-DON-ZEN-T2/HT2 using UB concetration class and three weights scenario. Lower and upper confidence interval of PDI were also reported.

MYCOTOXIN	WEIGHT	PDI UB	PDI LOWER CONFIDENCE INTERVAL	PDI UPPER CONFIDENCE INTERVAL
OTA	59.9	0.4172	0.4104	0.4240
FBs	59.9	23.2563	22.73637	23.77623
DON	59.9	0.48151	0.47207	0.49094
ZEN	59.9	0.07580	0.07251	0.07909
T2/HT2	59.9	0.24167	0.23521	0.24814
OTA	71.8	0.3494	0.3436	0.3550
FBs	71.8	19.65327	19.21803	20.08852
DON	71.8	0.41134	0.40335	0.41933
ZEN	71.8	0.06532	0.06256	0.06807
T2/HT2	71.8	0.20799	0.20258	0.21341
OTA	86.29	0.2920	0.2874	0.2968
FBs	86.29	16.14525	15.78367	16.50683
DON	86.29	0.34449	0.33776	0.35122
ZEN	86.29	0.05336	0.05102	0.05570
T2/HT2	86.29	0.16918	0.16472	0.17363

Table S8. PDI results for OTA-DON-ZEN using LB concetration class, three weights scenario for North and South Europe. Lower and upper confidence interval of PDI were also reported. * No needs for biomarker concentration data reconstruction, only MEAN class is available.

STUDY AREA	MYCOTOXIN	WEIGHT	PDI LB	PDI LOWER CONFIDENCE INTERVAL	PDI UPPER CONFIDENCE INTERVAL
SOUTH EU	OTA	59.9	0.2010	0.1944	0.2074
SOUTH EU	DON	59.9	0.22737	0.22302	0.23173
SOUTH EU	ZEN	59.9	0.06483	0.06480	0.06485
SOUTH EU	OTA	69.94	0.1746	0.1688	0.1806
SOUTH EU	DON	69.94	0.19531	0.19156	0.19907
SOUTH EU	ZEN	69.94	0.05552	0.05550	0.05555
SOUTH EU	OTA	81.9	0.1506	0.1456	0.1556
SOUTH EU	DON	81.9	0.16705	0.16385	0.17026
SOUTH EU	ZEN	81.9	0.04742	0.04740	0.04744
NORTH EU	OTA*	60.17	0.8088	0.7966	0.8212
NORTH EU	DON	60.17	0.33633	0.32969	0.34297
NORTH EU	ZEN	60.17	0.00176	0.00172	0.00179
NORTH EU	OTA*	73.00	0.6764	0.6662	0.6866
NORTH EU	DON	73.00	0.27699	0.27162	0.28236
NORTH EU	ZEN	73.00	0.00145	0.00143	0.00148
NORTH EU	OTA*	89.06	0.5498	0.5414	0.5580
NORTH EU	DON	89.06	0.23034	0.22587	0.23480
NORTH EU	ZEN	89.06	0.00122	0.00119	0.00124

Table S9. PDI results for OTA-DON-ZEN using MEAN concetration class, three weights scenario for North and South Europe. Lower and upper confidence interval of PDI were also reported. * No needs for biomarker concentration data reconstruction, only MEAN class is available.

STUDY AREA	MYCOTOXIN	WEIGHT	PDI MEAN	PDI LOWER CONFIDENCE INTERVAL	PDI UPPER CONFIDENCE INTERVAL
SOUTH EU	OTA	59.9	0.2184	0.2146	0.222
SOUTH EU	DON	59.9	0.28657	0.28185	0.29129
SOUTH EU	ZEN	59.9	0.06485	0.06483	0.06488
SOUTH EU	OTA	69.94	0.1874	0.1844	0.1906
SOUTH EU	DON	69.94	0.24309	0.23905	0.24712
SOUTH EU	ZEN	69.94	0.05553	0.05551	0.05556
SOUTH EU	OTA	81.9	0.1582	0.1556	0.1608
SOUTH EU	DON	81.9	0.20991	0.20644	0.21338
SOUTH EU	ZEN	81.9	0.04744	0.04742	0.04746
NORTH EU	OTA*	60.17	0.8088	0.7966	0.8212
NORTH EU	DON	60.17	0.58222	0.57839	0.58604
NORTH EU	ZEN	60.17	0.00177	0.00174	0.00181
NORTH EU	OTA*	73.00	0.6764	0.6662	0.6866
NORTH EU	DON	73.00	0.48282	0.4796	0.48603
NORTH EU	ZEN	73.00	0.00149	0.00146	0.00152
NORTH EU	OTA*	89.06	0.5498	0.5414	0.558
NORTH EU	DON	89.06	0.39378	0.39114	0.39641
NORTH EU	ZEN	89.06	0.00119	0.00117	0.00121

Table S10. PDI results for OTA-DON-ZEN using UB concetration class, three weights scenario for North and South Europe. Lower and upper confidence interval of PDI were also reported. * No needs for biomarker concentration data reconstruction, only MEAN class is available.

STUDY AREA	MYCOTOXIN	WEIGHT	PDI MEAN	PDI LOWER CONFIDENCE INTERVAL	PDI UPPER CONFIDENCE INTERVAL
SOUTH EU	OTA	59.9	0.2344	0.2256	0.2430
SOUTH EU	DON	59.9	0.30407	0.29801	0.31013
SOUTH EU	ZEN	59.9	0.12231	0.11989	0.12473
SOUTH EU	OTA	69.94	0.2024	0.1954	0.2092
SOUTH EU	DON	69.94	0.25879	0.25378	0.26380
SOUTH EU	ZEN	69.94	0.10588	0.10380	0.10795
SOUTH EU	OTA	81.9	0.1700	0.1640	0.1760
SOUTH EU	DON	81.9	0.22182	0.21754	0.22609
SOUTH EU	ZEN	81.9	0.08745	0.08573	0.08917
NORTH EU	OTA*	60.17	0.8088	0.7966	0.8212
NORTH EU	DON	60.17	0.63105	0.61421	0.64789
NORTH EU	ZEN	60.17	0.00195	0.00191	0.00199
NORTH EU	OTA*	73.00	0.6764	0.6662	0.6866
NORTH EU	DON	73.00	0.53178	0.51759	0.54597
NORTH EU	ZEN	73.00	0.00159	0.00156	0.00162
NORTH EU	OTA*	89.06	0.5498	0.5414	0.5580
NORTH EU	DON	89.06	0.43410	0.42238	0.44583
NORTH EU	ZEN	89.06	0.00131	0.00128	0.00134
